# Exquisite air sac histological traces in a hyperpneumatized nanoid sauropod dinosaur from South America

**DOI:** 10.1038/s41598-021-03689-8

**Published:** 2021-12-17

**Authors:** Tito Aureliano, Aline M. Ghilardi, Bruno A. Navarro, Marcelo A. Fernandes, Fresia Ricardi-Branco, Mathew J. Wedel

**Affiliations:** 1 Laboratory of Paleontology and Paleohidrogeology, Institute of Geosciences, University of Campinas (Unicamp), Campinas, Brazil; 2 Diversity, Ichnology and Osteohistology Laboratory (DINOlab), Department of Geology, Federal University of Rio Grande do Norte (URFN), Natal, Brazil; 3 Laboratório de Paleoecologia e Paleoicnologia (LPP), Departamento de Ecologia e Biologia Evolutiva (DEBE), Federal University of São Carlos (UFSCar), Sao Carlos, Brazil; 4 Laboratório de Paleontologia, Museu de Zoologia, University of Sao Paulo, Sao Paulo, Brazil; 5 College of Osteopathic Medicine of the Pacific and College of Podiatric Medicine, Western University of Health Sciences, Pomona, USA

**Keywords:** Respiration, Bone, Animal physiology, Palaeontology, Polarization microscopy, Histology, X-ray tomography, Palaeontology, Sedimentology

## Abstract

This study reports the occurrence of pneumosteum (osteohistological structure related to an avian-like air sac system) in a nanoid (5.7-m-long) saltasaurid titanosaur from Upper Cretaceous Brazil. We corroborate the hypothesis of the presence of an air sac system in titanosaurians based upon vertebral features identified through external observation and computed tomography. This is the fifth non-avian dinosaur taxon in which histological traces of air sacs have been found. We provided a detailed description of pneumatic structures from external osteology and CT scan data as a parameter for comparison with other taxa. The camellate pattern found in the vertebral centrum (ce) of this taxon and other titanosaurs shows distinct architectures. This might indicate whether cervical or lung diverticula pneumatized different elements. A cotylar internal plate of bone tissue sustains radial camellae (rad) in a condition similar to *Alamosaurus* and *Saltasaurus*. Moreover, circumferential chambers (cc) near the cotyle might be an example of convergence between diplodocoids and titanosaurs. Finally, we also register for the first time pneumatic foramina (fo) and fossae connecting camellate structures inside the neural canal in Titanosauria and the second published case in non-avian dinosaurs. The extreme pneumaticity observed in this nanoid titanosaur contrasts with previous assumptions that this feature correlates with the evolution of gigantic sizes in sauropodomorphs. This study reinforces that even small-bodied sauropod clades could present a hyperpneumatized postcranial skeleton, a character inherited from their large-bodied ancestors.

## Introduction

Dinosaurs developed a varied array of adaptations throughout more than 233 million years of evolution^1–9^. Postcranial skeletal pneumaticity (PSP) is one of the most remarkable adaptations shared by theropod and sauropod dinosaurs^10–17^. The pneumatic structures in the axial skeleton (e.g., foramina, fossae, and laminae) are a reflection of a permeating system of diverticula originating from the lungs^10,12,14,18–21^. Most of the approaches to studying PSP in dinosaurs were limited to the observation of either macroscopic structures or data from computed tomography (CT scans). More recently there have been approaches to detect PSP throughout the detection of pneumosteum, the histological correlates of the diverticula interaction with the bone tissue. However, few taxa have been sampled until now^22,23^. Therefore, there is an urge to sample more taxa across space and time.

Sauropods are often highlighted for their morphological adaptations to gigantism and increasingly high metabolic rates in derived clades^11,24–28^. Saltasaurid titanosaurians had already been highlighted for their hyperpneumaticity in the axial skeleton in comparison to other sauropodomorphs^11,12,29^. We sampled a posterior dorsal vertebra of an adult individual of a saltasaurid titanosaur from the Upper Cretaceous São José do Rio Preto Formation, Southeast Brazil. Computer tomography and histology were conducted throughout the neural arch and vertebral centrum to unveil the interaction between foramina, pneumatopores (pn), fossae, and their putative attached air sacs. Therefore, our results provide another piece for understanding the evolution of the respiratory system in derived sauropod dinosaurs.

## Material and methods

### Institutional abbreviations:

**CPPLIP**, Centro de Pesquisas Paleontológicas “Llewellyn Ivor Price”, Universidade Federal do Triângulo Mineiro, Peirópolis (Uberaba), Brazil; **DINOlab**, Dinosaur Ichnology and Osteohistology Laboratory, Federal University of Rio Grande do Norte, Natal, Brazil; Centro de Pesquisas Paleontológicas “Llewellyn Ivor Price”, Federal University of Triângulo Mineiro, Peirópolis (Uberaba), Brazil; **LPP-PV**, Laboratório de Paleoecologia e Paleoicnologia (UFSCar), Federal University of São Carlos (UFSCar), São Carlos, Brazil; **HU-UFSCar**, Hospital Universitário, Federal University of São Carlos, São Carlos, Brazil; **OMNH**, Oklahoma Museum of Natural History, Norman, Oklahoma; **PVL**, Paleovertebrate collection, Instituto “Miguel Lillo”, San Miguel de Tucumán, Argentina; **MCT**, Museu de Ciências da Terra, Rio de Janeiro, Brazil.

### Material

#### Specimen

**Figure 1:**
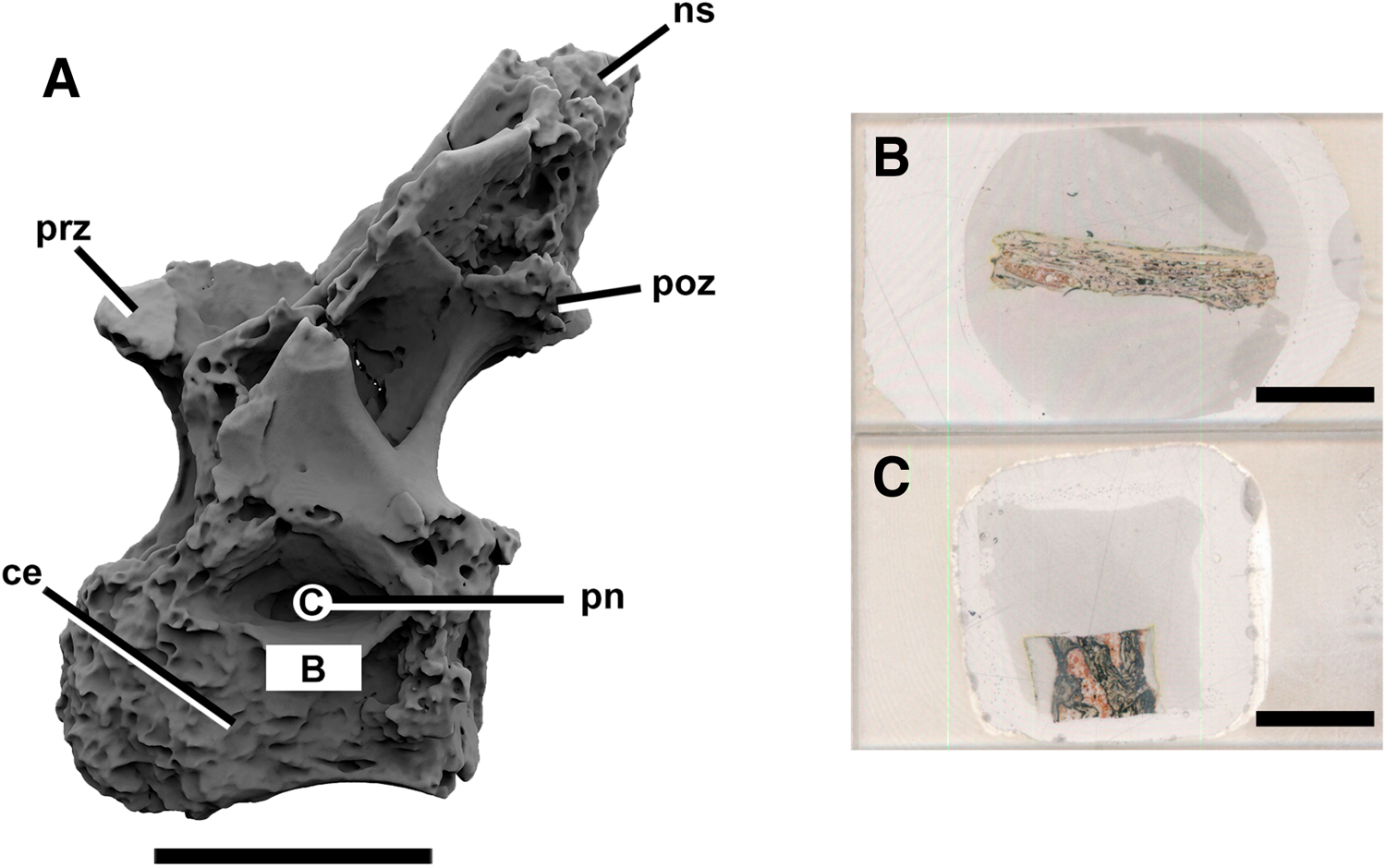
Posterior dorsal vertebra of the Upper Cretaceous nanoid saltasaurid LPP-PV-0200. Three-dimensional reconstruction from CT scan in left lateral view (**A**). Circle and rectangle show sampling planes and the respective thin sections are in (**B**,**C**). *ce* centrum, *ns* neural spine, *pn* pneumatopore, *poz* postzygaphophysis, *prz* prezygapophysis. Scale bar in (**A**) 10 cm; in (**B**,**C**) 1 cm. Computed tomography data processed with 3D Slicer version 4.10. Figures were generated with Adobe Photoshop CC version 22.5.1 X64.

The studied specimen (LPP-PV-0200; Fig. 1) corresponds to a posterior dorsal vertebra of a saltasaurid titanosaur. It was collected by Marcelo and Luciana Fernandes from the “Vaca morta” site^30^ on a farm at the Ibirá municipality, western São Paulo State, Southeast Brazil. Aline Ghilardi prepared and restored the specimen. It is part of the holotype of a nanoid titanosaur (5.7 m long) that is currently under description. We know from appendicular histology of the holotype that this was a senile individual. At least three specimens of this new taxon are known from the same stratum, and detailed results and discussion out of the scope of this paper will be published separately along the description of this new taxon. One of the specimens, also senile, showed pathologies associated with acute osteomyelitis and preserved phosphatized blood parasites inside the vascular canals^31^. However, the specimen we analyze in this research showed signs of neither pathologies nor parasitization. LPP-PV-0200 is housed at the Laboratory of Paleoichnology and Paleoecology at the Federal University of São Carlos, São Carlos city, São Paulo state, Brazil.

#### Locality and horizon

Ibirá municipality, São Paulo State, Upper Cretaceous São José do Rio Preto Formation (SJRP), Bauru Group, Southeast Brazil. The SJRP Formation is often regarded as Santonian in relative age^32–35^, and zircon dating of the underlying Adamantina Formation points to Coniacian-Santonian^36^.

### Anatomical nomenclature

For vertebral laminae and fossae, we followed^19,20,37^ respectively; For vertebral pneumatic structures, we followed the terminology of^9,12,14^. Histological terms are in accordance with standard literature^38,39^. Pneumosteum description followed^22,23^. The terms anterior and posterior were used instead of cranial and caudal, as suggested by^40^.

### Computed tomography imagery (CT scan)

A CT scan of the specimen was obtained before histological sampling using a Philips Diamond Select Brilliance CT 16-slice medical scanner with more than 200 slices and a voxel size of 0.75 mm at the HU-UFSCar. Acceleration voltage varied between 90 and 120 kV at a current of 367 mA. The methodology applied by^23^ was followed to analyze the data and generate the three-dimensional reconstruction with the software 3D Slicer version 4.10^41^ (available at https://www.slicer.org/). Raw data was uploaded to Morphobank platform and is available through this link: http://morphobank.org/permalink/?P4131. Figure organization and numbering follow^15^.

### Bone histology

Two histological samples were taken to track and describe the ‘pneumosteal bone’. Pneumosteum is a peculiar bone tissue type which forms the secondary trabeculae in postcranial bones that are pneumatized by diverticula of the respiratory system in saurischian dinosaurs^22^. Therefore, their presence indicates that specific areas were in contact with part of the lung-air sac system. The transversal section in the centrum ventral to the pneumatopore was produced following standard procedures^42^. The second sample was obtained from a core drill^43^ that crossed the entire vertebral centrum throughout the pneumatopores (see Fig. 1 for the planes of section). Thin sections were grounded to a thickness of ~ 40 to 50 µm. They were observed and photographed with a petrographic Leica DM750P microscope with coupled Leica EC3 camera, and imaging software Leica Application Suite (LAS) EZ version 1.6.0 X64 (available at https://www.leica-microsystems.com). Pictures were corrected for brightness and contrast, and composite images were generated with Adobe Photoshop CC version 22.5.1 X64 (available at https://www.adobe.com).

## Results

### CT scan of the dorsal vertebra LPP-PV-0200

**Figure 2:**
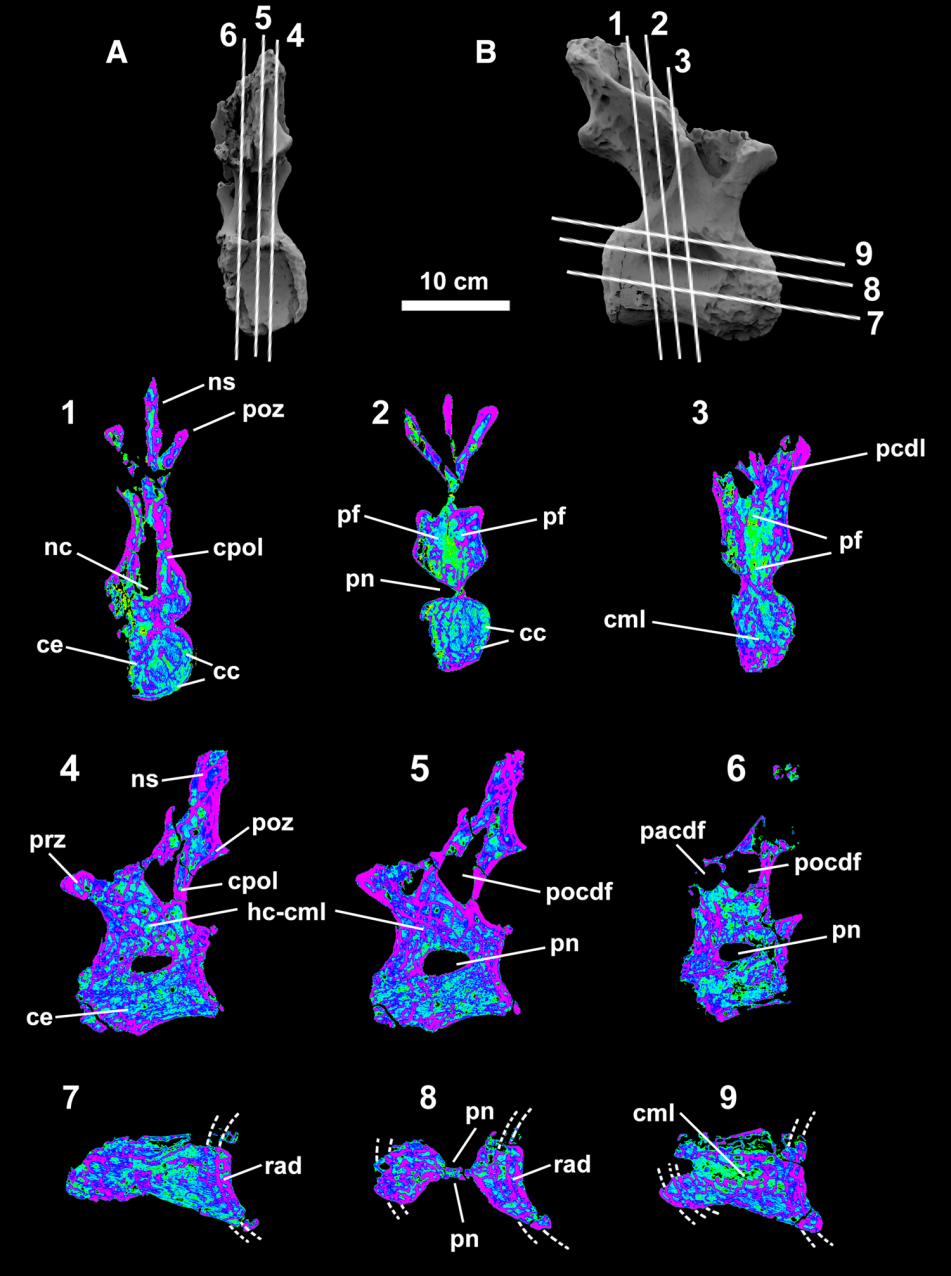
Dorsal vertebra internal structures of LPP-PV-0200. Reconstructed tomography model in distal (**A**) and right lateral (**B**) views illustrating subvertical tangential CT scan slices in false color (1–9). Images show that only a few structures had survived diagenesis which restricted the assessment of the internal architecture to limited spaces. Lighter blue and green indicate lower densities (e.g., pneumatic cavities). Purple and darker blue demonstrate denser structures (e.g., camellate bone). Dashed lines indicate internal plates of bone that sustain radial camellae. *ce* centrum, *cc* circumferential chambers, *cml* camellae, *hc-cml* ‘honeycomb’ camellae, *ns* neural spine, *pf* pneumatic foramen, *pn* pneumatopore, *pacdf* parapophyseal-centrodiapophyseal fossa, *pocdf* postzygapophyseal-centrodiapophyseal fossa, *rad* radial camellae. Computed tomography data processed with 3D Slicer version 4.10. Figures were generated with Adobe Photoshop CC version 22.5.1 X64.

**Figure 3:**
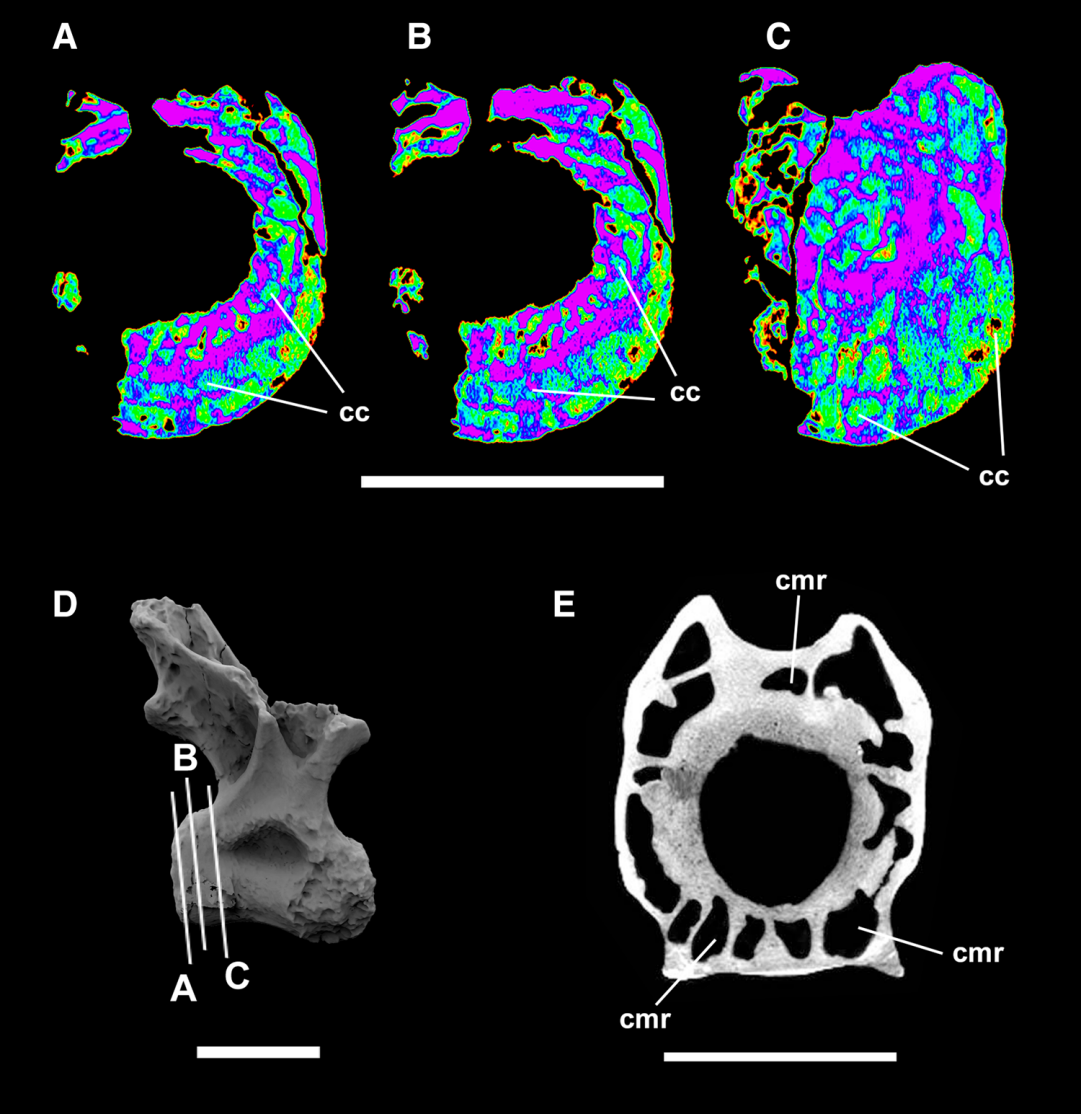
Dorsal vertebra centrum near the cotyle. Detail of internal structures of the saltasaurid titanosaur LPP-PV-0200 in A-C and comparison with *Apatosaurus* (OMNH 01094; from^47^) in (**E**). Reconstructed tomography model in lateral view (**D**) illustrating CT scan slices in false color (**A**–**C**). Small circumferentially-arranged chambers are present in LPP-PV-0200 near the cotyle. A similar condition has previously been documented in the camerate vertebra of *Apatosaurus* (**E**). *cc* circumferential chambers, *cmr* radially arranged camerae. Scale bar 10 cm. Computed tomography data processed with 3D Slicer version 4.10. Figures were generated with Adobe Photoshop CC version 22.5.1 X64.

Tomography slices allowed a 3D reconstruction of the saltasaurid dorsal vertebra (LPP-PV-0200; Figs. 1, 2). Internal bone architecture survived taphonomic processes and most of the pneumatic structures could be assessed (Fig. 2). In the vertebral centrum, there is an array of elongated parallel cavities extending dorsoventrally in anterior view (Fig. 2.1–3), and anteroposteriorly in lateral view (Fig. 2.4–5). Camellate architecture presents a general subtrapezoidal ‘honeycomb’ (hc-cml) arrangement (sensu^12^), especially in the lateral view of the neural arch. Camellate bone (cml) expands radially from the cotyle surface inwards (Fig. 2.7–9). Centropostzygapophyseal (cpol) and posterior centrodiapophyseal lamina (pcdl) laminae, prezygapophysis (prz), postzygapophyses (poz), and the neural spine (ns) present slightly thicker bone walls than the remaining structures. Camellae are smaller in the centrum (average width = 3.9 mm) but enlarged in the neural arch (average width = 4.8 mm). Pneumatopores are extremely deep on both sides, leaving only a thin bone wall (> 1 cm) in the fossae below the diapophysis. The entire centrum presents a ‘bow-tie’ shape ventrally in this cross-section (Fig. 2.8). Pneumatic foramina connect the neural canal to the inner camellate tissue laterally, ventrally, and dorsally (Fig. 2.2–3). Circumferential camellae are present around the cotyle rim (Fig. 3).

### Taphonomy and petrography

**Figure 4:**
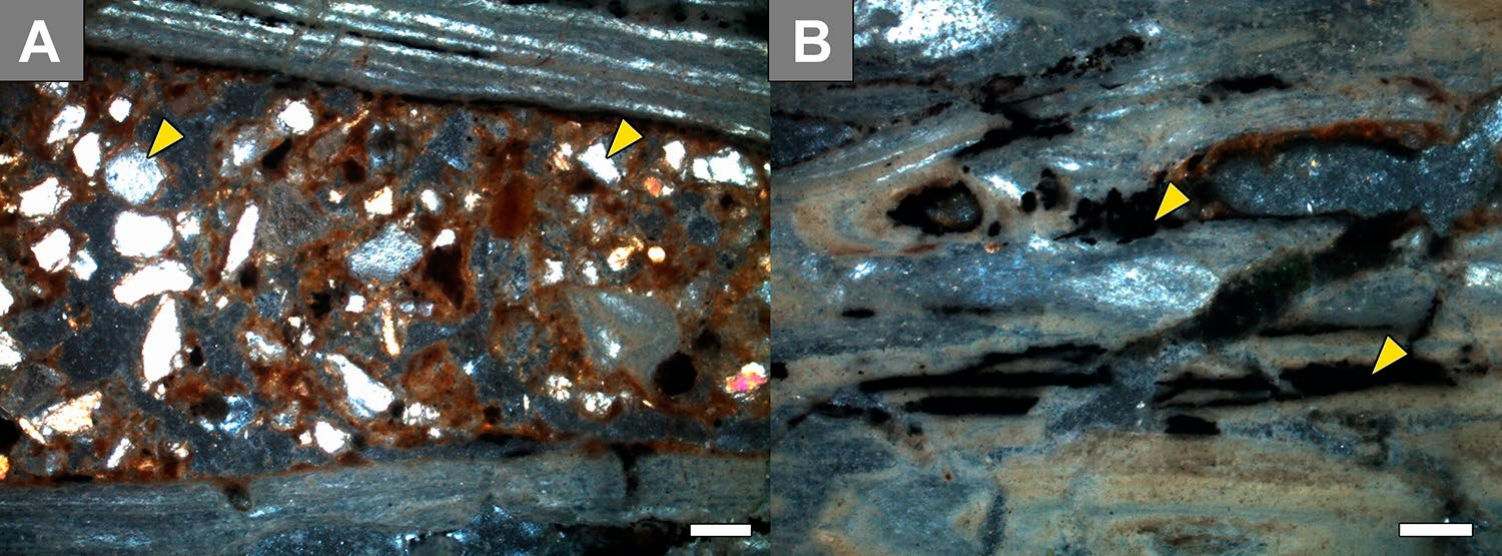
Taphonomic remarks in the microanatomy of LPP-PV-0200. (**A**) Poorly to moderately sorted subrounded to subangular mineral grains (arrow) and fragments of collapsed bone trabeculae (arrow) amidst the sedimentary matrix. Note the well-preserved birefringence of the bone apatite crystallites. (**B**) Opaque minerals infill the trabecular bone during weathering. All micrographs are in polarized light under crossed Nicols. Scale bar 100 µm. Photographs taken with Leica Application Suite (LAS) EZ version 1.6.0 X64. Figures were generated with Adobe Photoshop CC version 22.5.1 X64.

The specimen is fairly well preserved and there are no preparation marks on the surface, but the left lateral portion was slightly compromised during preburial transportation. Sedimentary mineral grains fill trabecular cavities (Fig. 4A). Thin opaque layers invade secondary osteons longitudinally in the medial shaft (Fig. 4B). These are early diagenetic features^44^. The birefringence of the bone apatite crystallites is well preserved and microstructure could be assessed.

### Histology

**Figure 5:**
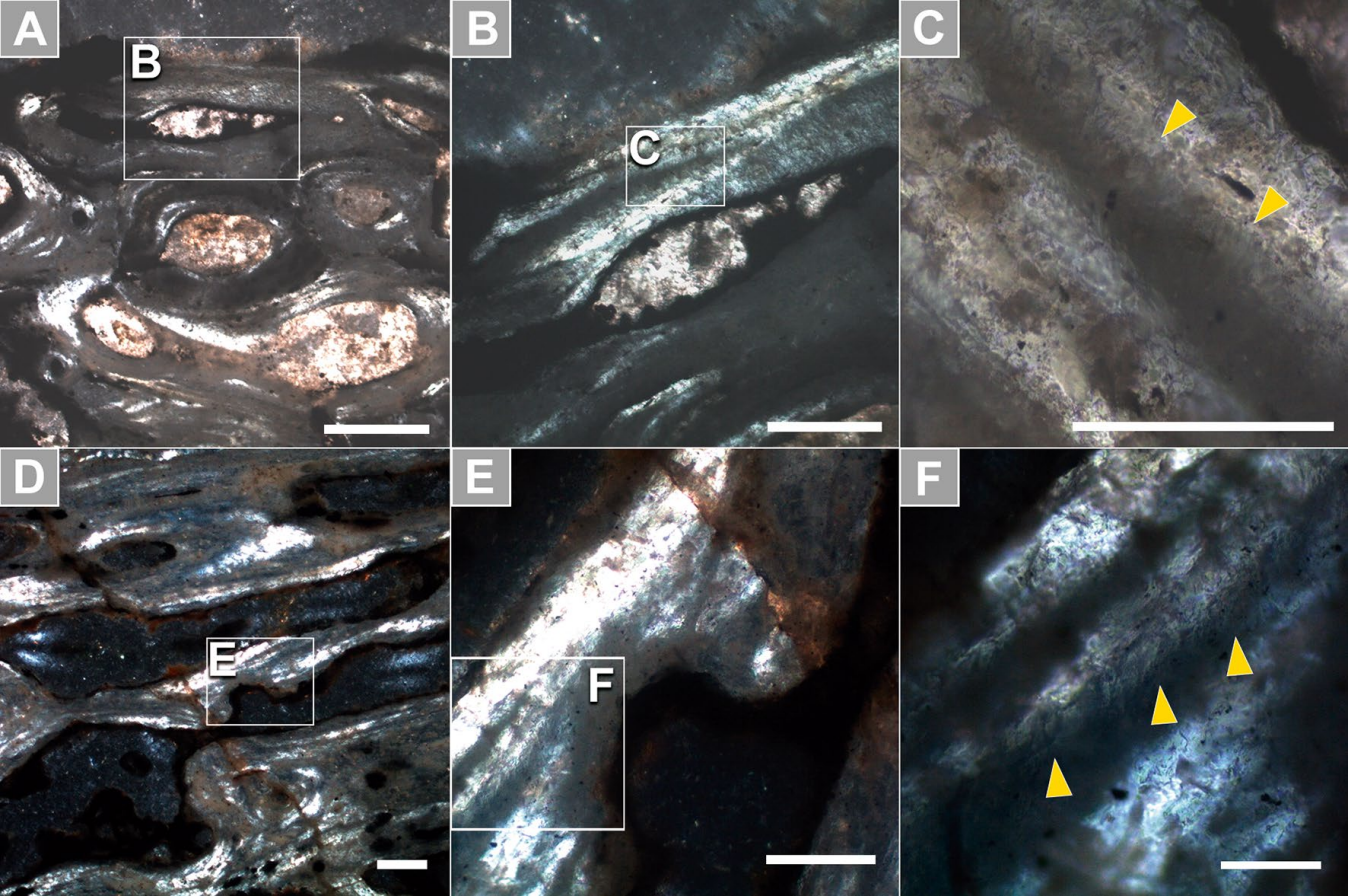
Occurrence of pneumosteum (arrows) in the posterior cervical vertebra of the saltasaurid titanosaur LPP-PV-0200. (**A**–**C**) Secondary bone in the internal centrum wall is comprised of pneumosteum entirely. (**D**–**F**) The trabeculae surrounding pneumatopores also consist solely of pneumosteum. Pneumosteal bone presents an undulose extinction and tiny asbestiform densely-packed ‘hair-like’ fibers. All micrographs are in polarized light under crossed Nicols. Scale bar in (**A**) 200 µm; in (**B**,**D**) 100 µm; in (**C**), (**E**) 50 µm; in (**F**) 20 µm. Photographs taken with Leica Application Suite (LAS) EZ version 1.6.0 X64. Figures were generated with Adobe Photoshop CC version 22.5.1 X64.

The thin sections of the vertebral laminae comprise bone trabeculae with visible camellate architecture (Fig. 5). Pneumosteum is widespread throughout the bone and comprises the secondary bone entirely. Pneumosteum is distinguished from regular trabeculae by comprising an array of tiny asbestiform densely-packed fibers (< 60 µm). Pneumosteal bone was found to be present both in centrum camellae (Fig. 5D,E) and the internal centrum wall (Fig. 5A–C).

## Discussion

Several pneumatic structures in the vertebra LPP-PV-0200 were associated with the organism’s respiratory system. The hyperpneumatization in this saltasaurid is evidenced throughout a complex arrangement of foramina, fossae, laminae, and camellate internal architecture. These structures once gave support to pulmonary diverticula, similar to the air sac system in extant birds. Tomography revealed a camellate architecture throughout most of the vertebral volume. The camellae are elongated in the vertebral centrum (anteroposteriorly and dorsoventrally), and slightly radial to the cotyle surface. A similar elongated (slightly radial) pattern has been observed in the cervical cotyles of *Austroposeidon* (MCT 1628-R^4^), and *Uberabatitan* (CPPLIP-1024^23^). Therefore, this camellate elongation may correlate to the structural needs of the vertebral articulation surfaces instead of being restricted to any specialized area (either cervical or dorsal). Additionally, camellate rings (perpendicular to the cotyle radial walls) have been proposed as possible ontogenetic markers as growth lines^4^. However, this feature is a non-linear indirect consequence of the changing diverticular and vascular architecture throughout ontogeny^17^, rather than linear growth marks as observed in appendicular cortical bones. An internal plate of bone tissue (laterally concave) under the cotyle sustains the radial camellae in LPP-PV-0200 (see Fig. 2.7–9). Such structure has also been observed in *Alamosaurus*^45^ and *Saltasaurus*^45,46^. Furthermore, LPP-PV-0200 shows at least two of these bony plates inside the cotyle of the vertebra, similar to the condition seen in *Alamosaurus*^45^ and different from *Saltasaurus*^46^ (just one plate). Such variations may be unrelated phylogenetically and could result from different developmental conditions during animal growth^17^. Another interesting feature is the presence of three of those bony plates inside the condyle, right below the neural spine (see Fig. 2.9). This condition has not been reported for any other taxon.

There are circumferential camellae around the margins of the centrum in cross-section, especially close to the cotyle (Fig. 3). Similar circumferential chambers were reported for *Apatosaurus* (OMNH 01094)^47^ but are seen for the first time in a fully camellate vertebra. Tissue architecture is rather chaotic closer to the cotyle in the basal Titanosauriformes *Giraffatitan*^48^. Therefore, this circumferentially-arranged small chambers in LPP-PV-0200 could be a convergence between diplodocoids and titanosaurs.

Table 1 resumes tissue organization in selected titanosaur vertebrae. The pneumatic architecture in the dorsal vertebral centrum of LPP-PV-0200 lacks the subtrapezoidal camellate disposition observed in *Austroposeidon* (MCT 1628-R; in distal midshaft view). This coincides with the pattern observed in a *Saltasaurus* dorsal (PVL 4017-17^11^). Coincidently, the subtrapezoidal pattern in *Saltasaurus* (PVL 4017-214) and in cervical vertebrae of the non-saltasaurid *Uberabatitan* might indeed correlate to the cervical air sac system. On the contrary, the chaotic pattern observed in LPP-PV-0200 and PVL 4017-214 might correlate with the thoracic or abdominal air sac systems. Here we define chaotic as an architecture with no regular arrangement/organization. However, more samples from different taxa are necessary to test if such differences in the bone tissue arrangement are indeed related to the surrounding air sac system, or if they result from other structural aspects of vertebral function throughout the axial series. The impact of ontogeny on the pneumatic architecture should also be considered when testing this hypothesis^17^.

**Table 1:** Camellate architecture in the presacral centra of titanosaurs.

Taxon	Specimen	Bibliography	Axial element	Bone tissue architecture (in cross-section)
*Saltasaurus loricatus*	PVL 4017–214	Cerda et al. (2012)^11^	Posterior cervical	Subtrapezoidal
	PVL 4017–47	Cerda et al. (2012)^11^	Middle dorsal	Chaotic
*Uberabatitan ribeiroi*	CPPLIP-1024	Aureliano et al. (2020)^23^	Posterior cervical	Subtrapezoidal
Saltasauridae from Ibirá locality	LPP-PV-0200	This article	Posterior dorsal	Chaotic

Pneumatic foramina and fossae are present in the neural canal both ventrally, dorsally, and laterally (see Fig. 2.2–3). Many birds have pneumatic foramina, fossae, or sculpted bone inside their neural canals. Pneumatic features inside the neural canal are osteological correlates of the supramedullary diverticula that run alongside or dorsal to the spinal cord, as observed in the CT scan of an ostrich neck^47^. It is known that supramedullary diverticula were present in at least some sauropods since such connections between the neural canal and pneumatic camellae were documented in *Giraffatitan*^48^. Nonetheless, here we describe these structures for the first time for Titanosauria; it is the second published case among all non-avian dinosaurs.

The extreme PSP in Argentinean saltasaurids has been previously reported^11,29^. This study in a nanoid saltasaurid from Brazil not only corroborates their studies with histological data but also reinforces their observation that PSP in sauropodomorphs does not always correlate solely with their giant sizes as previously hypothesized^12,24^.

## Conclusions

This study contributes with some insights for understanding the evolution of the respiratory system in dinosaurs. Our highlighted results are listed below.

- We expanded the occurrence of pneumosteum tissue to the saltasaurid titanosaur from the Upper Cretaceous Southeast Brazil. This also corroborates with the previous hypothesis that the pneumosteal bone tissue is a good signature for indicating the insertion of lung diverticula in the fossil record. This is the fifth non-avian dinosaur taxon in which histological traces of air sacs have been found.
- We provide a detailed description of PSP structures from the CT scan of a dorsal vertebra of the holotype. Hopefully, this will be a step towards standardizing comparison with other taxa. The camellate pattern found in the vertebral centrum of LPP-PV-0200 and shows distinct architectures from other titanosaurs. Further sampling in more taxa is necessary to test whether these differences in tissue organization are related to the surrounding air sac system or if they result from other aspects of vertebral function.
- Small circumferential chambers in this taxon may indicate a convergence between diplodocoids and titanosaurs.
- We documented for the first time pneumatic foramina and fossae connecting with camellate structures inside the neural canal in Titanosauria and the second published case in non-avian dinosaurs.
- The extreme pneumaticity observed in LPP-PV-0200 contrasts with previous assumptions that PSP correlates with giant sizes in dinosaurs. This study reinforces that even small-bodied sauropod clades could be hyperpneumatized. Nonetheless, this does not exclude the possibility that the early evolution of PSP in sauropods was correlated with the evolution of large body size*.*

At this point, only a few neosauropod taxa have been sampled for pneumosteum description in the literature. These include the diplodocoid *Diplodocus*, the basal macronarian *Europasaurus*, and the titanosaurs *Uberabatitan* and LPP-PV-0200. For future approaches, it would be relevant to expand sampling to basal sauropodomorphs and other sauropod clades.
